# Orthopædic Work in General Practice—I

**Published:** 1908-11-07

**Authors:** 


### Orthopædics.

### ORTHOPÆDIC WORK IN GENERAL PRACTICE—I.

No one has yet given a correct and successful definition of the word "orthopædics." Many definitions have been attempted, for orthopædics is as old as general surgery, and the term itself dates back to a period long antecedent to what we call the antiseptic era. For all that, the attempts have been poor things. Each of us may make his own definition, and though others may quarrel with him about its exactness, they cannot maintain that their endeavours are perfect and more worthy of acceptance. Thus we may say, with the Americans, that orthopædics mean the surgery of joints, or with the Germans, that it is the surgery of deformities. Both definitions are obviously untrue; they include in the one case too much, and in the other they do not include enough. A deformity of the eye hardly falls within the domain of the orthopædic surgeon, and again, there are conditions, such as wry neck, in which the orthopædist rightly interferes, though it cannot be proved that these are caused, primarily, by joint troubles. For that reason we shall not attempt to define what we mean by the word that titles this section, but we shall not be troubled if our readers take the broader line, and think of deformities, their correction and prevention, when the subject of orthopædics is discussed.

In private practice (and, unfortunately, too, in many of our hospitals) orthopædics is regarded as an unimportant branch of general surgery. Anyone, to judge by what happens daily at some hospitals, is fit to treat club feet, knock knees, and bad hips. When a special orthopædic branch is established it is placed under the charge of a surgeon who is on the general staff. In one case it is confided to the junior surgeon; in another it is treated as a small and insignificant branch that is hardly worth the attention it receives. There are, of course, brilliant exceptions, and the excellent orthopædic department at St. Bartholomew's Hospital needs no advertisement save that which it receives from the work it is doing. But there is a general tendency to decry orthopædics, and to refuse to recognise it as a specialty. This tendency, short-sighted and fallacious, is not met with in England alone, but is generally to be observed in Continental countries with the exception of Italy. It is true Germany, under the able leadership of the late Professor Hoffa, was roused for a time to a pitch of enthusiasm, and that Vienna has been the Mecca to the Medina of Berlin so far as the student of orthopædic surgery is concerned. But this sudden enthusiasm, so fruitful in its results and so encouraging in its spontaneousness, caused a violent reaction which is at present being felt both in Germany and Austria, and to a less extent in France. The general surgeon arose in his wrath and demanded the payment of Cæsar's dues to Cæsar. What had the orthopædic surgeon to do with nerve-grafting, forsooth? Or with webbed fingers and supernumerary digits? From time immemorial these have been privileges of the general surgeon. One may observe in passing that the period of time which the average person understands when he uses the adjective immemorial, hardly measures the age of nerve-surgery correctly, but that is a detail of fact, and such minutiæ do not trouble controversialists. At present the struggle is going on and is being keenly watched. The orthopædists will doubtless maintain their own, but they can only do so by bringing their work before the notice of the profession, and in securing the sympathy of the general practitioner. In Italy, which at present is in many ways a model for its neighbours so far as orthopædics is concerned, the speciality has already secured frank recognition. The magnificent institutions at Bologna and Milan, unrivalled in Europe and unsurpassed even by the American clinics, are proof of this fact.

We are here concerned not so much with the specialist's work as with orthopædics so far as it affects the general practitioner, and in the following articles an endeavour will be made to show how in private practice deformities can be adequately coped with. At the same time it is necessary to preface all remarks on the subject of orthopædics by stating that it is a specialty that is second to none in the amount of special skill, aptitude and resourcefulness it demands for its successful practice. A specialty in general practice is almost a contradiction in terms, and few general practitioners will dabble in eye surgery, for example. A knowledge of the essentials of the affection of the eye, ear, or throat, however, is of the greatest value in private practice. It not only enables the practitioner to detect and treat minor complaints, but it also renders it possible for him to recognise the first signs of dangers which he cannot cope with, and to send the patient for treatment to a colleague with more experience and greater operative skill than he himself possesses. This is a fact generally recognised. No one will send a patent suffering from an affection of the uterus to a general surgeon when a specialist on the surgery of the diseases of women is equally accessible; or a patient with cataract when the oculist is as close by. Yet it is almost the universal custom to take it for granted that general surgeons are also orthopædists, and that the man who can do an enterostomy with entire success is equally fit to operate on a case of talipes calcaneus *in sensu strictiori*. There can be no greater mistake than that, and orthopædics will gain when the general practitioner comes to admit that to act in such a fashion is to lose sight of certain factors that are necessary for success. It is unfortunate that orthopædics is treated in such a step-motherly fashion as it is. The average hospital student, for instance, hears little about it, and has few chances of seeing good successful work. He learns to look upon half successes as triumphs, and to regard the maximum of efficiency as unattainable. The result is that he is incapable of judging the harm that is done by bad operating and injudicious attempts —the meddlesome surgery still favoured by many general surgeons where orthopædics is concerned.

